# Human Herpesvirus-6 Pneumonitis around the Engraftment of Cord Blood Transplantation following Foscarnet Prophylaxis in a Patient with Acute Leukemia

**DOI:** 10.1155/2015/949265

**Published:** 2015-01-11

**Authors:** Takashi Ishio, Tomoyuki Endo, Kohei Okada, Akio Shigematsu, Satoshi Hashino, Takanori Teshima

**Affiliations:** Department of Hematology, Hokkaido University Hospital, Kita-15 Nishi-7, Kita-ku, Sapporo, Hokkaido 060-0815, Japan

## Abstract

Human herpesvirus-6 (HHV-6) reactivation is sometimes observed in immunocompromised patients, especially after allogeneic stem cell transplantation. The complications of HHV-6 reactivation in this setting are mainly recognized as HHV-6 encephalitis. We herein report the case of a patient who developed HHV-6 pneumonitis after cord blood transplantation (CBT). A 35-year-old male underwent CBT for T-cell/myeloid mixed phenotype acute leukemia and achieved neutrophil engraftment on day 31. He had received foscarnet as prophylaxis for HHV-6 reactivation. A computed tomography (CT) scan to evaluate the leukemic tumor showed bilateral interstitial pneumonitis on day 33, although he had no respiratory symptoms. The findings of the CT scan were consistent with those of HHV-6 pneumonitis that were reported previously. HHV-6 DNA, but no other pathogens, was detected in his bronchoalveolar lavage (BAL) fluid. The patient was successfully treated with a therapeutic dose of foscarnet. This case indicates that performing a CT scan around the time of neutrophil engraftment can play an important role in detecting the early phase of HHV-6 pneumonia, and BAL should be considered if features consistent with HHV-6 pneumonitis are observed in patients with a risk of HHV-6 reactivation.

## 1. Introduction

Human herpesvirus-6 (HHV-6) infection generally occurs early in life. At the age of 5 years, more than 90% of children are seropositive for the virus, and by 17 years of age, 98% are positive [1]. Immunocompromised patients, especially patients who have undergone allogeneic hematopoietic stem cell transplantation (HSCT), are at increased risk of the reactivation of HHV-6 [2]. The complications of HHV-6 reactivation in the post-HSCT setting are mainly recognized as HHV-6 encephalitis. In contrast to encephalitis, HHV-6 pneumonitis is very rare, although an association between HHV-6 infection and pneumonitis was previously suggested [3]. We herein report the case of a patient with T-cell/myeloid mixed phenotype acute leukemia who developed HHV-6 pneumonitis after cord blood transplantation (CBT).

## 2. Case Report

A 35-year-old Japanese male presented with generalized lymphadenopathy and an excess of blasts in his peripheral blood. He was diagnosed to have T-cell/myeloid mixed phenotype acute leukemia by a bone marrow examination. He received remission induction therapy and consolidation therapy. These provided a complete remission. We considered HSCT to be an appropriate treatment for him, because the leukemic tumor had infiltrated into the liver, spleen, and pericardium at the time of the diagnosis. As we could not identify a human leukocyte antigen- (HLA-) matched related or unrelated donor, we chose umbilical cord blood as the graft source. A CBT (nucleated cell count 2.33 × 10^7^ cells/kg, CD34-positive cell count 0.99 × 10^5^ cells/kg, HLA allele two-locus mismatch, female donor) was performed after administering a myeloablative conditioning regimen consisting of medium dose etoposide, cyclophosphamide, and total body irradiation [4]. The graft-versus-host disease (GVHD) prophylaxis comprised tacrolimus and short-term methotrexate.

**Figure 1:**
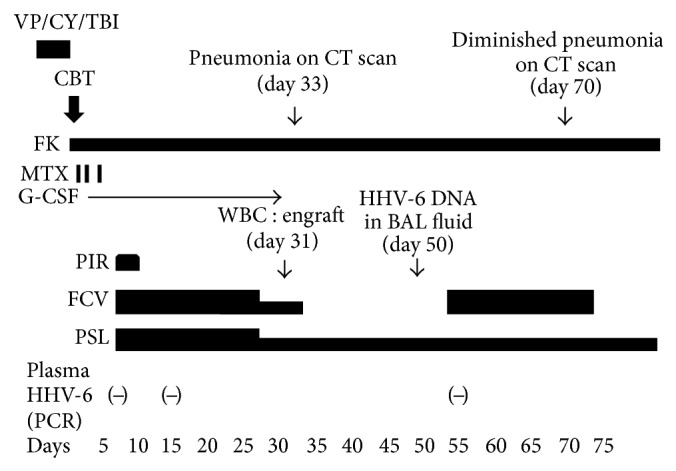
Clinical course of the present case. VP: etoposide, CY: cyclophosphamide, TBI: total body irradiation, CBT: cord blood transplantation, FK: tacrolimus, MTX: methotrexate, G-CSF: granulocyte colony-stimulating factor, PIR: preengraftment immune reaction, WBC: white blood cell, BAL: bronchoalveolar lavage, FCV: foscarnet, and PSL: prednisolone.

The following narrative describes the patient's treatment after CBT (Figure 1). Nine days after transplantation, the patient developed a fever, skin rash, and retention of fluid. He was diagnosed to have a preengraftment immune reaction (PIR) [5], and corticosteroid treatment was initiated, which resulted in improvement of the PIR. As PIR after CBT was considered to be a risk factor for HHV-6 encephalitis [6], foscarnet (90 mg/kg/day) was administered from day nine for prophylaxis of HHV-6 reactivation. HHV-6 DNA was not detected by a polymerase chain reaction (PCR) assay in his serum on days 7 and 15, and a physical examination revealed no significant findings relative to encephalitis. He achieved neutrophil engraftment on day 31, and the foscarnet was discontinued.

**Figure 2:**
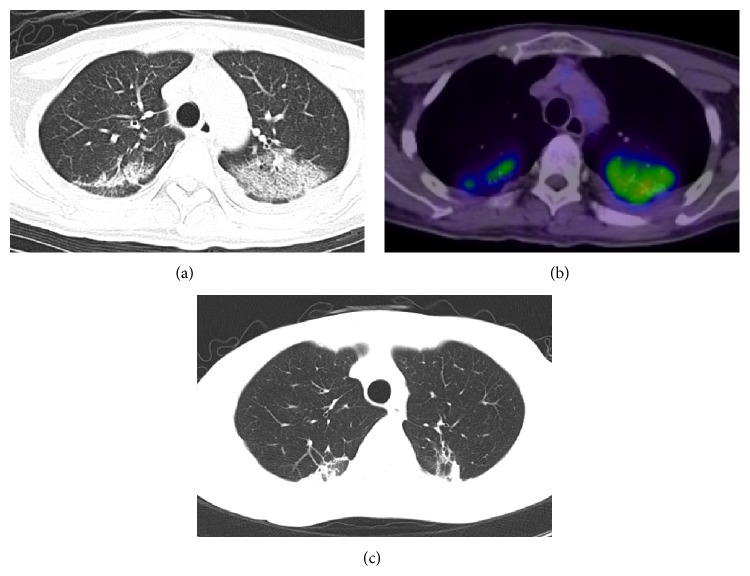
(a) HHV-6 pneumonitis. The data shown here was acquired after CBT. CT scan on day 33 showed reticulation, ground-glass opacity, consolidation, and peripheral lung sparing in bilateral lung fields. (b) FDG-PET scan on day 41 revealed a maximum standard uptake value of 6.0 in bilateral lung fields. (c) HHV-6 pneumonitis was diminished after foscarnet therapy as assessed by CT scan on day 82.

A computed tomography (CT) scan to evaluate the leukemic tumor showed bilateral interstitial pneumonitis on day 33 (Figure 2(a)), although the patient had no respiratory symptoms or significant findings of hemogasanalysis. A fluorodeoxyglucose positron emission tomography (FDG-PET) scan also showed abnormal uptake, with a maximum standard uptake value of 6.0 on day 41 (Figure 2(b)). It was suggested that the pneumonitis was active. Infection, GVHD, and drug-induced pneumonitis were considered for the differential diagnosis. A physical examination revealed no significant findings relative to GVHD, and the pneumonitis was refractory to antibiotics, antifungal drugs, and intravenous immunoglobulin. Therefore, a bronchoalveolar lavage (BAL) examination was performed on day 50 to detect the pathogen causing the interstitial pneumonitis. The BAL fluid consisted of 88% macrophages, 11.1% lymphocytes, and 0.8% neutrophils and did not include whole blood. HHV-6B DNA was detected (6 × 10^3^ copies/mL) in the BAL fluid using a PCR assay, while other pathogens, including HHV-6A, adenovirus, parainfluenza virus, cytomegalovirus (CMV), Epstein-Barr virus (EBV), respiratory syncytial virus (RSV), herpes simplex virus, rhinovirus, and metapneumovirus,* Mycoplasma*,* Aspergillus*,* Pneumocystis jiroveci*,* Legionella pneumophilia*, and* Chlamydia pneumonia* were not detected by PCR assays, and no bacteria or fungi were detected by bacterial or mycology cultures of the BAL fluid. The findings of the CT scan were consistent with that of HHV-6 pneumonitis reported previously [7, 8], and the findings of the FDG-PET scan indicated that the pneumonitis was active. Hence, the patient was diagnosed to have HHV-6 pneumonitis. Magnetic resonance imaging (MRI) of the brain showed no findings of HHV-6 encephalitis. Treatment with foscarnet (180 mg/kg/day) was initiated on day 54, although HHV-6 DNA was not detected in the serum. The pneumonitis was improved on day 70 as assessed by CT scan, and foscarnet was discontinued on day 74. Since then, there have been no findings of recurrence of the HHV-6 pneumonitis (Figure 2(c)).

## 3. Discussion

To date, there have been several reports of pneumonitis considered to be associated with HHV-6 after HSCT [9–14]. In almost all of them, provisional diagnoses of HHV-6 pneumonitis were made by detecting plasma HHV-6 DNA. However, there have been few case reports of HHV-6 pneumonitis after HSCT in which HHV-6 was directly detected in the BAL fluid [7, 8, 15].

Although standard diagnostic criteria for HHV-6 pneumonitis have not been established, BAL plays an important role in diagnosis [9]. CT scanning also played a role in the diagnosis of HHV-6 pneumonitis in the present case. The CT images of HHV-6 pneumonitis in the previously published cases showed reticulation, ground-glass opacity, consolidation, and peripheral lung sparing in bilateral lung fields [7, 8]. These findings were consistent with those of the present case. They also showed septal thickening, pulmonary fibrosis with bronchiectasis, and centrilobular nodules with a tree-in-bud appearance [7, 8]. Although the radiographic findings of HHV-6 pneumonitis are nonspecific, CT scanning is useful for the early detection of pneumonitis. FDG-PET scanning is being increasingly used to assess the metabolic activity of pulmonary inflammatory cells [16]. FDG-PET scanning will therefore be a valuable imaging technique for the management of patients with pneumonitis, as it can show the activity of the pneumonitis, which cannot be detected by CT scans. In the present case, we could assume that the interstitial shadow on CT was active pneumonitis based on the findings of the FDG-PET scan, even though he had no respiratory symptoms.

In the reported cases, including this provisional one, the clinical course of the HHV-6 pneumonitis varied from mild to severe. Some were improved by treatment with foscarnet [7, 8, 15], but others were refractory to such therapy and sometimes required mechanical respiratory support, which was attributed to adult respiratory distress syndrome [17]. Carrigan et al. reported that active HHV-6 replication in the BAL fluid might be an indication of the need for curative therapy [15]. All reported cases of HHV-6 pneumonitis which were diagnosed by BAL followed a good clinical course. From this point of view, a prompt and confirmed diagnosis by BAL seems to be important for ensuring the adequate treatment of HHV-6 pneumonitis.

There is compelling evidence to implicate the following risk factors in HHV-6 reactivation: an allogeneic stem cell transplant source, leukemia or lymphoma as an underlying disease, the use of a corticosteroid or immunosuppressant, and the existence of GVHD, PIR, and engraftment syndrome (ES). HHV-6 is more frequently reactivated after transplantation from unrelated donors, especially after CBT [6, 18–23]. In our present case, all of the above risk factors applied except for GVHD and ES.

The primary treatment for HHV-6 reactivation is the use of foscarnet or ganciclovir. However, HHV-6 reactivation is reported to develop in the early stage after HSCT [7, 8, 15], and this was also true in our present case. As treatment with ganciclovir in the early stage after HSCT may give rise to prolonged neutropenia, foscarnet seems to be a more appropriate treatment.

The prophylactic use of foscarnet for patients considered to be at high risk for HHV-6 reactivation has been tried to prevent HHV-6 encephalitis [24]. However, the optimal dose of foscarnet for prophylaxis has not been established. Ishiyama et al. suggested that the prophylactic use of foscarnet at 90 mg/kg/day might reduce the risk of HHV-6 encephalitis [24]. On the other hand, Ogata et al. reported that 50 mg/kg/day of foscarnet did not effectively suppress HHV-6 reactivation [25]. In our case, HHV-6 pneumonitis developed during prophylactic use of foscarnet (90 mg/kg/day). Treatment with a therapeutic dose (180 mg/kg/day) of foscarnet may reduce the reactivation of HHV-6; however, this is not always feasible after transplantation because of its renal toxicity. Further investigations are needed to establish safe and effective prophylaxis against HHV-6 reactivation. At this time, the most important approach for high-risk patients seems to be the early detection of reactivation of the HHV-6 and prompt treatment.

To detect the reactivation of HHV-6, the plasma HHV-6 DNA level has often been checked in high-risk patients. Ishiyama et al. reported that thrice-weekly monitoring of the plasma HHV-6 DNA level might allow for preemptive therapy [26]. Typically, HHV-6 DNA becomes positive in the plasma from 14 to 27 days after transplantation, and HHV-6-related disease develops close to the time of neutrophil engraftment [23]. However, the plasma HHV-6 DNA level was reported to change dynamically from negative to a high level during a short period of time [23]. Therefore, it will not necessarily be easy to detect an increase of the virus, especially if the DNA level is only checked every few days. In the present case, the plasma HHV-6 DNA level was only checked on days 7, 15, and 55, and the findings were all negative. We speculated that the level might have been positive if we had checked at other intervals, especially around the time of neutrophil engraftment.

We were able to detect the findings of interstitial pneumonitis by CT scan around the time of neutrophil engraftment in the present case, and these findings led to a further examination, the diagnosis of HHV-6 pneumonitis, and appropriate treatment. From our experience, performing a CT scan around the time of neutrophil engraftment is thought to be useful as the first step in the early diagnosis of HHV-6 pneumonitis. If features consistent with HHV-6 pneumonitis are observed, BAL should be strongly taken into consideration in patients at high risk for HHV-6 reactivation, even if they have no respiratory symptoms.

In conclusion, we herein reported an uncommon complication of HHV-6 reactivation in a patient with acute leukemia after CBT. This case indicates that CT scans and BAL play important roles in the diagnosis of HHV-6 pneumonitis in HSCT recipients.
